# Case Report: Multimodality evaluation and clinical management of a single coronary artery

**DOI:** 10.3389/fcvm.2023.1295602

**Published:** 2024-01-08

**Authors:** Patrick McAlpin, Matthew Purlee, Ann Dickey, Arun Chandran, Mohammad Ahmad Zaki Al-Ani

**Affiliations:** ^1^ Division of Cardiovascular Imaging, Department of Radiology, University of Florida, Gainesville, FL, United States; ^2^ Lake Erie College of Osteopathic Medicine-Bradenton, Bradenton, FL, United States

**Keywords:** coronary artery anomaly (CAA), single coronary artery, interarterial, intramural, pediatric

## Abstract

A 14-year-old male with no significant medical history presented with intermittent palpitations for 2–3 months that occurred at rest and were associated with light-headedness. Electrocardiogram in clinic showed sinus arrhythmia with early repolarization and no ischemic changes. The echocardiogram showed normal cardiac structure and function, however, there was a concern for possible anomalous origin of the left coronary artery. Contrast-enhanced CT coronary artery angiogram confirmed a single coronary origin from the right coronary sinus. The single main coronary artery gave rise to the right coronary artery (RCA) and the left coronary artery (LCA). The LCA demonstrated a trans-septal course before it gave rise to the left anterior descending and left circumflex artery. There were intraarterial and intramural portions of the LCA, and the sinoatrial node artery arose from the LCA. The RCA demonstrated a normal course to the right atrioventricular groove, and the posterior descending artery arose from the RCA. Treadmill exercise stress test showed excellent functional capacity without exercise-induced chest pain or ischemic ECG changes. Invasive coronary angiography ruled out luminal narrowing or dynamic compression. Given the absence of physiologic or anatomic evidence of coronary flow restriction, no intervention was pursued and the palpitations were deemed to be likely unrelated to the coronary anomaly and eventually subsided spontaneously on 6 month follow-up.

## Introduction

A coronary artery anomaly is a rare anatomic variant of the coronary system with a range of clinical significance from those patients who remain asymptomatic to others predisposed to major cardiac events, especially sudden cardiac death when in the presence of certain, high-risk morphological features. Therefore, when coronary artery anomalies are suspected, a thorough clinical evaluation with the concomitant use of multi-modality imaging is essential to guide clinical management. In this report, we detail the case of a 14-year-old male who presented with palpitations and was later diagnosed with a single coronary artery arising from the right coronary sinus with certain unusual features.

## Presentation

**Figure 1:**
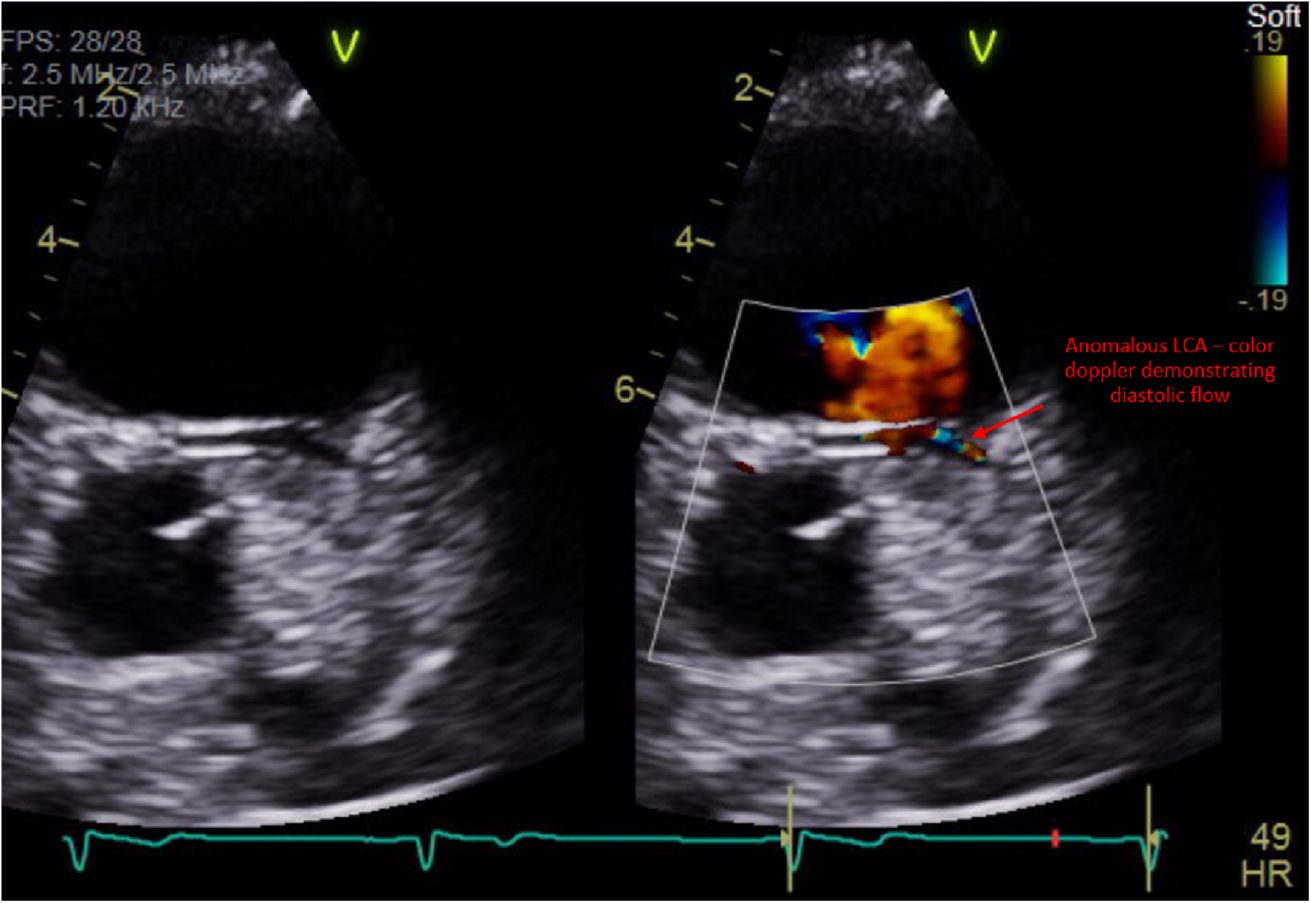
Transthoracic echocardiogram demonstrating diastolic flow via color Doppler within a vessel that was concerning for a left coronary artery with an anomalous origin.

A 14-year-old male with no significant medical history presented with intermittent palpitations for 2–3 months that occurred at rest and were associated with light-headedness. Electrocardiogram in clinic showed sinus arrhythmia with early repolarization and no ischemic changes. The echocardiogram showed normal cardiac structure and function, however, there was a concern for possible anomalous origin of the left coronary artery (Figure 1).

**Figure 2:**
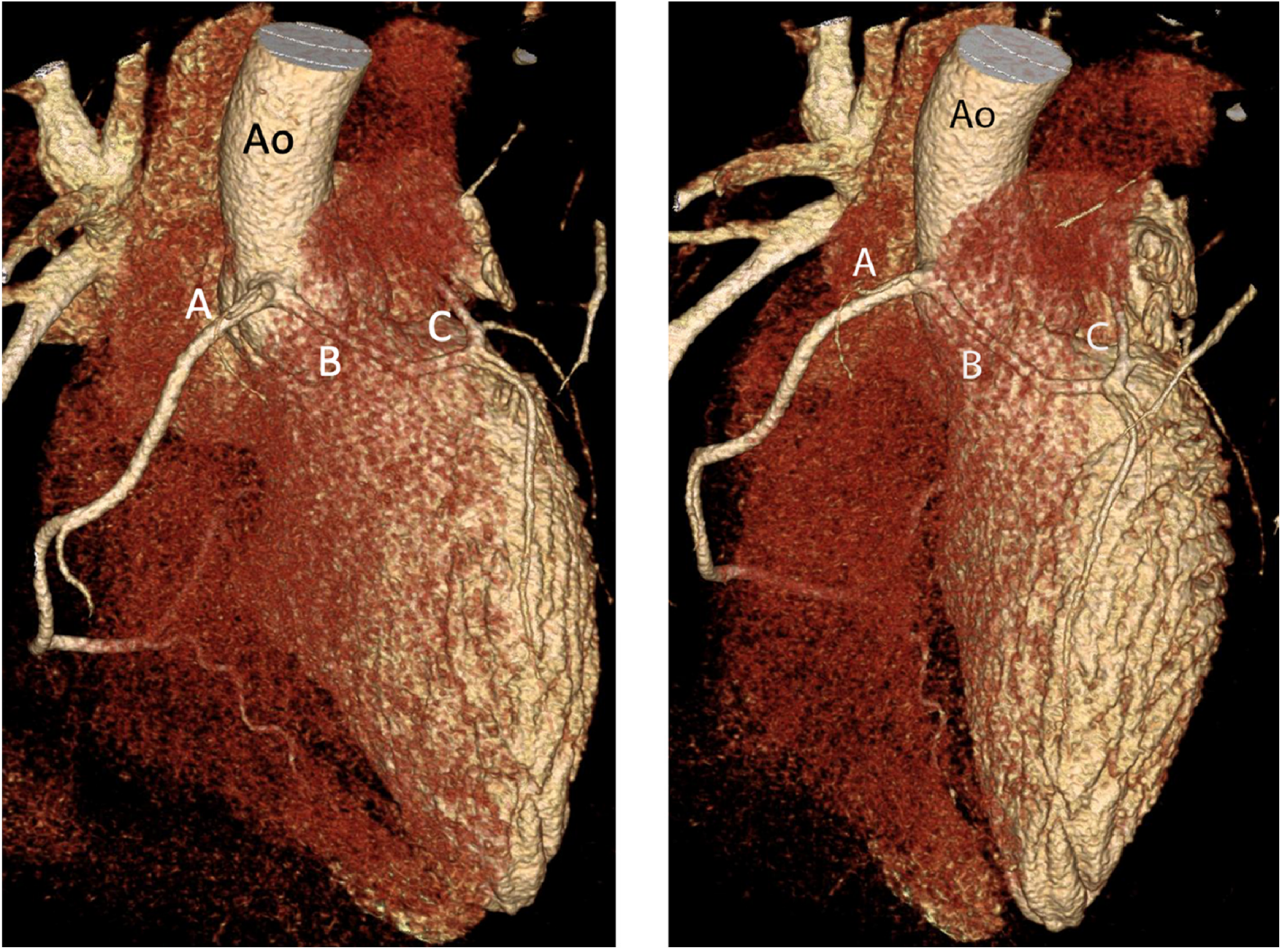
An oblique (left) and anteroposterior (right) projection of a volume-rendered 3D reconstruction of the heart demonstrating a single CA dividing into the RCA (**A**) and LCA (**B**) with subsequent trans-septal course of the LCA (**C**) Ao, aorta.

**Figure 3:**
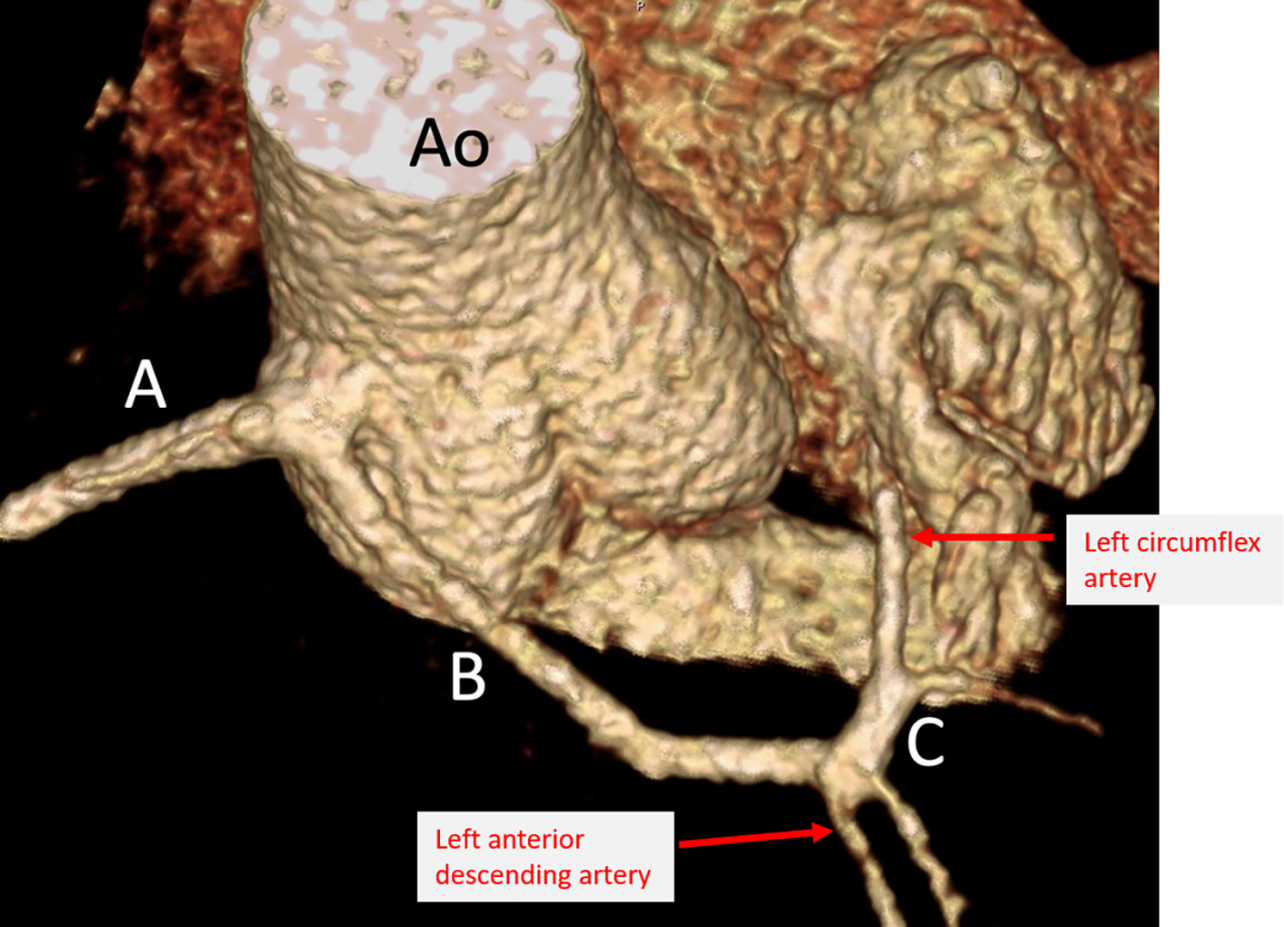
Selective view of a volume-rendered 3D reconstruction demonstrating single coronary artery dividing into RCA (**A**) and LCA (**B**) with subsequent trans-septal course of the LCA (**C**). Ao, aorta.

**Figure 4:**
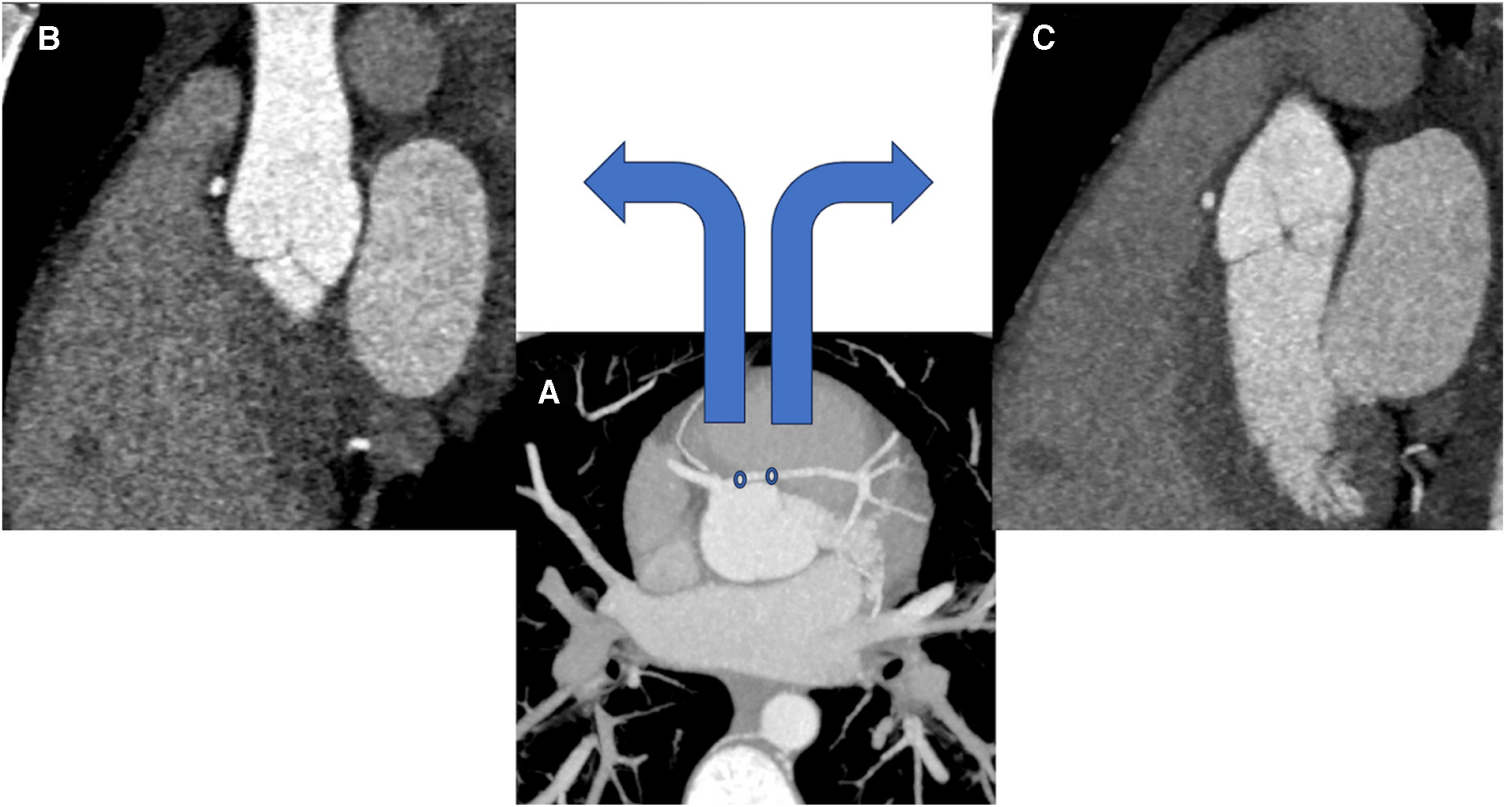
(**A**) is a coronary CTA displaying the course of the single coronary artery as it arises from the right coronary sinus in an axial plane with maximal image projection. (**B**) corresponds to the first circle of the proximal LCA seen on (**A**) and displays its interarterial course – note the presence of mediastinal fat surrounding the LCA. (**C**) corresponds to the second circle of the LCA seen on (**A**) and displays its intramural course. Images were obtained at early diastole.

Contrast-enhanced CT coronary artery angiogram confirmed a single coronary origin from the right coronary sinus. The single main coronary artery gave rise to the right coronary artery (RCA) and the left coronary artery (LCA). The LCA demonstrated a trans-septal course before it gave rise to the left anterior descending and left circumflex artery (Figures 2, 3). There were interarterial and intramural portions of the LCA, and the sinoatrial node artery arose from the LCA (Figure 4). The RCA demonstrated a normal course to the right atrioventricular groove, and the posterior descending artery arose from the RCA. Treadmill exercise stress test showed excellent functional capacity without exercise-induced chest pain or ischemic ECG changes. Invasive coronary angiography ruled out luminal narrowing or dynamic compression. Given the absence of physiologic or anatomic evidence of coronary flow restriction, no intervention was pursued and the palpitations were deemed to be likely unrelated to the coronary anomaly and eventually subsided spontaneously on 6 month follow-up.

## Discussion

The term “coronary artery anomaly” refers to a broad spectrum of various configurations of the typical coronary artery system. An anomaly is defined as any coronary pattern with a feature that is not commonly encountered, whether it refers to the ostium, course, branching pattern, etc (1). Coronary artery anomalies occur in <1% of the general population (1). The coronary artery anomaly may be clinically silent, however there is a spectrum of clinical sequelae attributed to coronary artery anomalies that includes chest pain, sudden death, cardiomyopathy, syncope, dyspnea, ventricular fibrillation, and myocardial infarction (2). There are multiple classification schemes in the literature that stratify the rarity and clinical significance of each anomaly (3). Generally, coronary artery anomalies can be classified into three groups that include anomaly of origination and course, anomaly of intrinsic coronary arterial anatomy, and anomaly of coronary termination (4, 5). A single coronary artery, as seen in this case, is exceedingly rare as it occurs in 0.0024%–0.044% of the population and carries a high risk for sudden death (5). A single coronary artery may arise from either the left or right coronary sinus, and its course may demonstrate features that are risk factors for clinically significant symptoms, such as a course with interarterial and/or intramural portions (1). The most frequent type of clinically significant variant anatomy is the interarterial course of the anomalous coronary artery with abnormal origin from the opposite sinus of Valsalva (5). An interarterial course means the coronary artery courses between the pulmonary arterial trunk and the aorta, which when present there may be constriction of the anomalous coronary artery during intense physical activity, leading to decreased blood flow (3, 6). Therefore, this anatomic feature carries a higher risk of sudden cardiac death among the previously mentioned complications (7). Additional risk factors of a sudden cardiac death include an acute angle of take-off, an intramural course of the anomalous coronary artery, and a crevice-like ostium (6, 8).

There are multiple studies in the literature that support the risk of sudden cardiac death with coronary artery anomalies, with a reported range of 11.8%–19% of deaths in young athletes in the US being related to coronary artery anomalies (9, 10). Screening efforts have been proposed given the risk of sudden cardiac death in this population, in addition to military personnel who undergo strenuous activity, each with their respective limitations. Such screening methods include echocardiography, contrast-enhanced electron-beam tomography, magnetic resonance imaging, and coronary angiography (1). Echocardiography, transthoracic or transesophageal, with Doppler interrogation is the initial modality choice for evaluating an anomalous coronary artery when clinically suspected. If at least two normally located coronary ostia are identified with echocardiography, no further workup is likely required (11). If further imaging is required, contrast-enhanced CT or MRI may be performed. Contrast-enhanced CT offers excellent spatial resolution and identifies most anomalies of coronary course with drawbacks including the use of ionizing radiation and contrast agents that may be potentially nephrotoxic or allergenic (12). MRI offers the advantage of no ionizing radiation or potentially harmful contrast agents with the added benefits of great visualization of the coronary artery origins, especially in patients with congenital defects (13). The main limitation of MRI is the poor visualization of the distal coronary artery course (13). Finally, coronary angiography is a strong modality for diagnosis of an anomalous coronary artery, especially when compared to echocardiography. One study demonstrated a 0.17% incidence of an anomalous coronary artery arising from the opposite sinus, while coronary angiography demonstrated a 1.07% incidence (11). The drawback of coronary angiography is that it is a more invasive exam, though (11).

There is a range of opinions regarding a trans-septal course of a LCA from a single RCA with a recent study pointing to a benign course while a paper from 2018 revealed that these patients could pose a high-risk variant (14, 15). The difficulty however lies in the surgical approach to the trans-septal course since potentially approaching it with an un-roofing procedure would involve exposure of a long trans-septal course, which carries a higher incidence potential for post-operative stenotic complications. Coronary un-roofing is performed for CAAs with an intramural segment, where the wall between the intramural segment and the aorta is unroofed creating a wide neo-ostium (14). Un-roofing carries an additional risk of aortic insufficiency given the risk of damaging the intercoronary commissure. Accordingly, the threshold for surgical indication in this particular variant is high, and since this patient had no specific ischemic symptoms or signs on imaging, a more conservative approach to management was taken.

Demonstration of the clinical significance of an anomalous coronary artery, i.e., myocardial ischemia, is challenging as standard clinical submaximal stress-test protocols are often inadequate to provide definitive information (1). Stress-perfusion tests (CT or MR) can delineate any myocardial ischemia associated with CAAs, which can be nuanced in certain cases and difficult to link to clinical significance. However, the presence of large perfusion defects certainly helps guide management toward a more invasive route, such as in cases of pulmonary origin of coronary arteries and coronary atresia (1). Long-term Holter monitoring is considered to rule out ventricular tachycardia that may be induced by ischemia (1). Most individuals are asymptomatic for long periods of time with the discovery of an anomaly as a result of a diagnostic work-up prompted by an atypical chest-pain syndrome (14). This clinical course contrasts with the individuals who die suddenly, commonly at a young age and after strenuous activity (14). Conservative treatment that includes observation and use of pharmaceutical therapy is often employed in cases of asymptomatic individuals (14). For those who are at high risk of sudden cardiac death, surgical treatment, such as coronary reimplantation or CABG, should be considered (3). Should be noted, however, CABG is of limited utility in pediatric patients who will likely outlast their bypass grafts, while reimplantation is a technically challenging surgery requiring an appropriate take-off angle when transferring the CAA ostium to its correct sinus (14).

Coronary artery anomalies exist across a broad spectrum of both anatomic configuration and clinical presentation, often appearing incidentally on imaging obtained for other indications. Providers need to be able to recognize the differences between clinically significant and benign CAAs, which requires the use of multimodality imaging ranging from echocardiography to invasive angiography. Examples of major features of CAAs that are associated with clinical significance include intramural and interarterial segments of the anomalous coronary artery. Once the underlying anatomy is defined, stress tests can be performed among other options to help delineate prognostic information. Depending on the information acquired during the clinical investigation, management may require simple observation vs. surgical intervention. Proper recognition and risk stratification of CAAs will ultimately lead to improved outcomes in this patient population and continued research into this ever-growing subject.

## Data Availability

The original contributions presented in the study are included in the article/Supplementary Material, further inquiries can be directed to the corresponding author.
